# Preliminary Pilot Assessment of TSPAN32 Detectability in Peripheral Blood as a Potential Circulating Biomarker in Advanced-Stage Nasopharyngeal Carcinoma Patients Undergoing Chemoradiation

**DOI:** 10.3390/cimb48101003

**Published:** 2026-09-29

**Authors:** Nadia Ayu Mulansari, Rafika Indah Paramita, Septelia Inawati Wanandi, Aru Wisaksono Sudoyo, Marlinda Adham Yudharto

**Affiliations:** 1 Doctoral Program in Biomedical Sciences, Faculty of Medicine, Universitas Indonesia, Central Jakarta 10430, Indonesia; nadia.mulansari@ui.ac.id; 2 Hematology–Medical Oncology Division, Department of Internal Medicine, Faculty of Medicine, Universitas Indonesia, Central Jakarta 10430, Indonesia; 3 Department of Medical Chemistry, Faculty of Medicine, Universitas Indonesia, Central Jakarta 10430, Indonesia; 4 Bioinformatics Core Facilities—The Indonesian Medical Education and Research Institute, Faculty of Medicine, Universitas Indonesia, Central Jakarta 10430, Indonesia; 5 Department of Biochemistry and Molecular Biology, Faculty of Medicine, Universitas Indonesia, Central Jakarta 10430, Indonesia; 6 Molecular Biology and Proteomics Core Facilities—The Indonesian Medical Education and Research Institute, Faculty of Medicine, Universitas Indonesia, Central Jakarta 10430, Indonesia; 7 Department of Ear, Nose, and Throat Clinic, Faculty of Medicine, Universitas Indonesia, Central Jakarta 10430, Indonesia

**Keywords:** nasopharyngeal carcinoma, molecular markers, differentially expressed genes, *TSPAN32*, bioinformatics, transcriptomics

## Abstract

Nasopharyngeal carcinoma (NPC) is highly prevalent in Southeast Asia, including Indonesia, with most patients presenting at advanced clinical stages. Reliable, minimally invasive circulating biomarkers for monitoring treatment response are lacking. This study aimed to identify candidate blood-based transcriptomic biomarkers for advanced-stage NPC through integrative bioinformatic analysis and to evaluate the expression dynamics of a prioritised candidate gene, *TSPAN32*, in peripheral blood of patients undergoing chemoradiation. Differentially expressed genes (DEGs) were identified from the GEO microarray dataset GSE53819 (18 advanced-stage NPC tissues vs. 18 non-cancerous nasopharyngeal tissues) using GEO2R. Candidate DEGs were prioritised through protein–protein interaction (PPI) network analysis (STRING confidence ≥ 0.9), functional enrichment analysis (GO via Enrichr; KEGG pathway via ShinyGO v0.82), and survival analysis (Kaplan–Meier). Overlapping DEGs were identified against a previously published blood-based NPC transcriptomic dataset with 11 early-stage NPC patients, 11 advanced-stage NPC patients, and 11 healthy donors. Analysis of GSE53819 identified 943 upregulated and 1503 downregulated DEGs (|log_2_FC| ≥ 1; Padj < 0.05). Functional enrichment of upregulated DEGs was dominated by inflammatory response and cytokine activity pathways, while downregulated DEGs were enriched in B-cell activation and immunoglobulin receptor binding. Protein–protein interaction network construction (STRING confidence ≥ 0.9) and hub gene identification using the MCC algorithm identified six candidate genes among the downregulated DEGs: *ADRA2A*, *SELP*, *TSPAN32*, *PEAR1*, *P2RX1*, and *DGKG*. Kaplan-Meier survival analysis demonstrated that lower expression of *SELP* (HR = 0.57; *p* = 0.00053), *TSPAN32* (HR = 0.56; *p* = 0.00025), and *P2RX1* (HR = 0.48; *p* = 1.2 × 10^−5^) was significantly associated with shorter overall survival in head and neck carcinoma, supporting *TSPAN32* as the primary candidate for clinical validation. RT-qPCR examination demonstrated that *TSPAN32* transcript abundance in peripheral blood was significantly upregulated following chemoradiation in nine of ten patients (mean Log_2_FC = 2.08 ± SD 1.09, 95% CI 1.29–2.86; one-sample *t*-test t(9) = 6.01, *p* = 0.0002; Wilcoxon signed-rank test *p* = 0.002). Primer specificity for *TSPAN32* and *ACTB* was confirmed by melt-curve analysis and *in silico* validation (Primer-BLAST, OligoAnalyzer, UCSC In-Silico PCR). RBC transfusion status did not significantly modulate the magnitude of *TSPAN32* expression change (independent-samples *t*-test, t = 1.86, *p* = 0.101). This integrative study identifies *TSPAN32* as a candidate circulating biomarker responsive to chemoradiation in advanced-stage NPC. These exploratory findings require confirmation in larger, prospective, longitudinal cohorts before clinical translation.

## 1. Introduction

Nasopharyngeal cancer, or nasopharyngeal carcinoma (NPC), is a malignant epithelial tumor located in the nasopharynx and is the most common malignant tumor in the ear, nose, and throat (ENT) area [1,2]. The causes of NPC are complex and not fully understood at this time, but carcinogenesis may occur due to genetic, environmental, and viral factors, particularly Epstein–Barr Virus (EBV) infection [3]. The prevalence of NPC varies greatly geographically. For example, in non-endemic areas such as the Americas and Europe, NPC is extremely rare, occurring at less than 1 case per 100,000 people [4]; meanwhile in Asia, specifically certain parts of China, the incidence rate is much higher, namely more than 21 cases per 100,000 people, contributing to around 18% of all types of cancer. NPC cases in the Asian population are predominantly in middle age, the fourth to sixth decades of life, whereas in Africa, specifically in areas where EBV infection is endemic, the majority of cases occur in children [3].

According to the Global Cancer Observatory (GLOBACAN), 83.3% of NPC cases and 83.6% of deaths occur in Asia, with the highest incidence rates in East Asia (52.6%), Southeast Asia (35.8%), and South-Central Asia (9.2%). Nine of the ten countries with the highest age-standardized incidence rate (ASIR) in the world are in Southeast Asia. Indonesia itself ranks second in the world for NPC incidence after China (18.8%) and has the third-highest ASIR (6.1%) [5,6]. One of the biggest challenges in managing NPC in Indonesia is that patients often come when their complaints get worse. Several studies show that the majority of patients (51–70.5%) come to the hospital with advanced-stage NPC, III-IV [7,8,9]. The behavior of patients who have just arrived at the final stage is usually caused by non-specific NPC symptoms such as nasal congestion, epistaxis, and a mass in the neck, which can be considered a benign condition [10].

Treatment for advanced NPC consists of chemotherapy and radiotherapy, typically with platinum agents combined with intensity-modulated radiotherapy (IMRT). This combination is known to be the mainstay of treatment because it is associated with high rates of local control [11]. Since this regimen is myelosuppressive and frequently induces anemia, red blood cell (RBC) transfusion is a standard supportive therapy [12,13]. Transfusion aims to correct anemia and potentially improve tumor oxygenation, thereby increasing radiosensitivity [13,14].

In advance-stage NPC, clinical management also becomes very challenging due to the variability in clinical presentation and the difficulty in linking predictions of disease progression to therapeutic outcomes. In recent years, biomarkers have been used as important monitoring tools in NPC management [15]. There are at least two general categories of NPC biomarkers: EBV-related biomarkers and cellular biomarkers. EBV-related biomarkers include EBNA1, LMP1/2, EBER 1/2, and BART miRNAs [16]. These biomarkers provide insight into the underlying tumor biology, aid in assessing prognosis, and inform treatment decision-making [15]. However, these biomarkers have several limitations. For example, in treatment monitoring, EBV biomarkers generally show little change, even after curative therapy [17]. In addition, not all NPC patients show detectable EBV reactivation and the lack of standardization in EBV DNA testing protocols, including detection equipment, DNA extraction methods, target DNA fragments, quality control, and especially threshold determination, remains a controversial issue [16,18]. Thus, these limitations indicate an unmet need for the development of more accurate biomarkers, particularly in assessing NPC disease progression.

In the past two decades, the emergence of omics technologies has brought about major changes in the understanding of biological systems. One such technology is transcriptomics, which studies gene expression in RNA, revealing which genes are expressed or inhibited under certain conditions [19]. Transcriptomic analysis is an excellent tool in the biomarker discovery process because it allows for comprehensive identification of gene expression patterns related to tumorigenesis, progression, and therapeutic response [16,20]. This approach allows the identification of unique gene expression patterns specific to a particular tumor, offering deeper insights into disease pathogenesis and potential therapeutic targets [16,19]. One of the most frequently used instruments in the field of transcriptomics is a repository called the Gene Expression Omnibus (GEO). GEO is a public functional genomics data repository created by the National Center for Biotechnology Information (NCBI) that archives and freely distributes high-throughput microarray and RNA-seq transcriptomic datasets [21]. In the context of transcriptomic research on NPC, most studies have focused on relatively more invasive tissue specimen collection and are limited in long-term serial monitoring during therapy [22,23]. This study aims to identify candidate biomarkers detectable through tissue and blood examination in advanced-stage NPC patients using the bioinformatics analysis approach. 

## 2. Materials and Methods

### 2.1. Study Design

This study combines primary clinical samples from NPC patients at Cipto Mangunkusumo Hospital and secondary datasets from the GEO dataset that was conducted between September 2023 and March 2026. The study used a two-phase integrated biomarker discovery and clinical study design. Phase 1 included an *in silico* bioinformatic discovery phase that used publicly accessible transcriptome datasets to identify potential DEGs linked with advanced-stage NPC. Phase 2 was a prospective examination phase in which the expression of the key candidate gene was measured using RT-qPCR in peripheral blood samples obtained longitudinally from a prospective cohort of advanced-stage NPC patients undergoing chemoradiation. A healthy control comparator group was not included in the RT-qPCR examination cohort.

### 2.2. Study Population and Ethics

This study comprised two phases. The *in silico* bioinformatic analysis of publicly available transcriptomic datasets (GSE53819 and the referenced blood transcriptomic dataset) began in September 2023. The prospective clinical component, comprising peripheral blood sample collection and RT-qPCR examination in advanced-stage NPC patients, was conducted under institutional ethics approval (KET-219/UN2.F1/ETIK/PPM.00.02/2024).

Adult patients aged over 18 with histopathologically confirmed advanced-stage nasopharyngeal carcinoma (clinical stage III–IV) scheduled to undergo concurrent chemoradiation as initial therapy were deemed eligible for inclusion.

Inclusion criteria required complete medical records encompassing histopathological data as well as a willingness to submit and sign informed consent. Patients were excluded if they had (i) started chemoradiation before enrolling; (ii) clinically significant comorbidities that could confound blood transcriptomic profiles (active autoimmune disease, concurrent haematological malignancy, known primary immunodeficiency); or (iii) insufficient blood sample volume for RNA extraction at either time point.

### 2.3. Clinical Treatment and Blood Sample Collection

Patients were classified into transfusion and non-transfusion groups during chemoradiation. All groups of patients undergoing chemoradiation were collected 5 mL of blood into an EDTA tube for analyses requiring plasma or cellular components before undergoing chemoradiation and after chemoradiation, with a maximum time of up to 8 weeks.

A standardized research data sheet form or case report form for recording subject identification codes, demographic data, clinical characteristics, histopathological diagnosis, tumor stage, metastatic status, treatment history, RBC transfusion status and volume, hemoglobin level/anemia status, EBV status, and laboratory results.

### 2.4. RNA Extraction and Quality Control

Whole blood was centrifuged at 2500× *g* for 10 min at 25 °C. The buffy-coat layer was separated and used for RNA extraction. RNA was extracted using the Quick-RNA™ MiniPrep Plus kit (Cat. No. R1058, Zymo Research, Irvine, CA, USA) according to the manufacturer’s protocol. RNA purity was assessed using the 260/280 nm absorbance ratio on the Implen NanoDrop P300 (Implen, Munich, Germany). RNA concentration was measured using the Qubit 3.0 Fluorometer (Thermo Fisher Scientific, Waltham, MA, USA) and the Qubit RNA Assay Kit (Thermo Fisher Scientific, Waltham, MA, USA). For biomarker validation, the primer pair was designed using Primer-BLAST (https://www.ncbi.nlm.nih.gov/tools/primer-blast/ (accessed on 27 May 2025) and evaluated using OligoAnalyzer Tools (https://sg.idtdna.com/pages/tools/oligoanalyzer (accessed on 27 May 2025) and UCSC *In Silico* PCR (https://genome.ucsc.edu/cgi-bin/hgPcr (accessed on 27 May 2025). The target-gene expression was normalized to ACTB (β-actin) as the housekeeping gene. RNA was reverse-transcribed into cDNA using Vazyme HiScript III RT SuperMix for qPCR with following steps: RNA template 1 μL was mixed with 4xgDNA wiper 4 μL, and ddh2O 11 μL into pcr tube, the sample in pcr tube was qPCR was incubated in 42 °C for 2 min in thermo cycler (First step of PCR).

Preparation for reverse transcription was conducted by adding 5x Hiscript III qRT Supermix and PCR product from the first step. Subsequently, the sample was loaded into a thermo cycler with incubation temperature 37 °C for 15 min and 85 °C for 5 s (Second step of PCR). The qPCR reaction contained 1 μL cDNA at 15 ng/μL, 10 μL of 2× Vazyme SYBR Green PCR Master Mix, 0.8 μL each of forward and reverse primers at the concentration reported in the laboratory protocol, and ddH2O 8.2 μL to a final volume of 20 μL. Amplification was performed with these following conditions: initial denaturation 95 °C for 30 s, cycling reaction 95 °C for 10 s and 60 °C for 30 s, and melting curve 95 °C for 15 s, 60 °C for 60 s, and 95 °C for 15 s. All results were recorded and documented.

### 2.5. In Silico DEG Discovery Phase

#### 2.5.1. Dataset Selection and DEG Identification

The DEGs biomarkers were analyzed by using the datasets available in Gene Expression Omnibus (GEO) database. The keyword “nasopharyngeal cancer” was entered to search the appropriate datasets. GSE53819 was used to compare 18 advanced-stage NPC tissues and 18 non-cancerous nasopharyngeal tissues. The platelet RNA-sequencing study by Yuanji Xu et al., comprising 11 early-stage NPC patients, 11 advanced-stage NPC patients, and 11 healthy donors, was used as a blood-based source of DEGs (Table S1) [24]. Several publicly available NPC transcriptomic datasets were reviewed during study, including GSE12452 and GSE118613, these were not used in the present analysis as they comprise different NPC stage compositions (e.g., mixed-stage or non-advanced-stage tumors) that did not match the advanced-stage-specific focus of this study.

Raw GEO data were processed using GEO2R. An adjusted *p*-value < 0.05 was used as the statistical threshold. For DEG selection, an absolute log2 fold change of at least 1 was applied; for miRNA profiling, the fold-change criterion reported in the analysis was at least 1.5. Upregulated and downregulated genes from tissue and blood sources were compared using Venny 2.1.0. Overlapping candidates were selected for further biological, survival, and molecular validation analyses.

The GSE53819 DEGs were entered into STRING for PPI analysis using a confidence score of 0.9. Networks were visualized in Cytoscape software (version 3.8.2) [25]. Molecular complex detection (MCODE) algorithm included a degree cutoff of 2, k-core/k-score of 2, maximum depth of 100, and node score cutoff of 0.2. Gene Ontology enrichment was analyzed using ENRICHR [26,27,28], KEGG pathway [29,30,31] enrichment was analyzed using ShinyGO version 0.82 [32], and downregulated miRNA sets were analyzed using TAM 2.0 [33].

#### 2.5.2. Protein–Protein Interaction (PPI) Network Analysis

DEGs passing threshold criteria were submitted to the STRING database (v11; https://string-db.org/ (accessed on 21 May 2025)) [34] for protein–protein interaction (PPI) network construction. It seeks to facilitate the investigation of protein networks and their relationships with diverse biological processes. A minimum interaction confidence score of 0.9 (high confidence) was applied to ensure network quality. The resulting network was visualised using Cytoscape v3.8.2 [25]. Network clustering was performed using the Molecular COmplex DEtection (MCODE) algorithm with parameters: degree cut-off = 2, k-score = 2, maximum depth = 100, and node score cut-off = 0.2 [20].

#### 2.5.3. Functional Enrichment Analysis

Gene ontology (GO) enrichment analysis was performed using Enrichr (https://maayanlab.cloud/Enrichr/ (accessed on 23 May 2025)) [26,27,28] covering Biological Process (BP) and Molecular Function (MF) categories. KEGG pathway [29,30,31] enrichment analysis was conducted using ShinyGO v0.82 (https://bioinformatics.sdstate.edu/go/ (accessed on 23 May 2025)) [32]. The significance threshold of Padj < 0.05 was applied for all enrichment analyses.

#### 2.5.4. Survival Analysis

The prognostic significance of candidate DEGs was evaluated using Kaplan–Meier survival analysis with respect to overall survival (OS) in head and neck carcinoma, using the KM-Plotter tool (https://kmplot.com/ (accessed on 25 May 2025)) [35]. Log-rank *p*-values and hazard ratios (HR) with 95% confidence intervals were calculated. A significance threshold of *p* < 0.05 was applied. Only DEGs showing statistically significant OS associations were considered for downstream cross-referencing and experimental clinical analysis.

#### 2.5.5. Cross-Reference with Blood-Based Transcriptomic Dataset

To identify DEGs that are concordantly expressed in both NPC tissue and peripheral blood, the GSE53819 DEGs were overlapped with the published blood-based NPC transcriptomic dataset reported by Yuanji Xu et al. [24]. Identical thresholds (|log_2_FC| ≥ 1; Padj < 0.05) were applied to both datasets to ensure comparability. Overlap was visualised using Venn diagram analysis (Venny 2.1.0; https://bioinfogp.cnb.csic.es/tools/venny/ (accessed on 26 May 2025)) [36]. Genes concordantly dysregulated across both compartments were considered candidate circulating biomarkers, as their detectability in accessible peripheral blood reflects the transcriptomic aberrations observed in NPC tissue.

Overlap between tissue-derived and blood-derived DEGs was defined as genes meeting the significance threshold (|log_2_FC| ≥ 1, Padj < 0.05) in both the GSE53819 tissue analysis and the reference peripheral blood transcriptomic dataset. Given the modest size of both source datasets, this comparison was expected to yield a limited number of overlapping candidates and was used as a prioritisation step.

### 2.6. RT-qPCR Examination

#### 2.6.1. Primer Design

The candidate for biomarker reference sequence was obtained from NCBI (Gene ID: 10077). Primers for RT-qPCR amplification were designed using Primer-BLAST (https://www.ncbi.nlm.nih.gov/tools/primer-blast/ (accessed on 27 May 2025)) [37] and quality-checked using the IDT OligoAnalyzer Tool (https://sg.idtdna.com/pages/tools/oligoanalyzer (accessed on 27 May 2025)) [38] and the UCSC *In Silico* PCR tool (https://genome.ucsc.edu/cgi-bin/hgPcr (accessed on 27 May 2025)) [39]. Beta-actin (*ACTB*) was selected as the housekeeping gene reference following confirmation of stable expression across clinical samples, consistent with established practice in peripheral blood gene expression studies.

#### 2.6.2. RT-qPCR and Relative Quantification

Complementary DNA (cDNA) synthesis was performed from 100 ng total RNA per sample using Vazyme HiScript III RT SuperMix for qPCR. RT-qPCR reactions were performed in duplicate technical replicates on each sample. Thermal cycling conditions and reaction composition followed standard protocols for SYBR Green-based detection. *ACTB* (β-actin) was used as the endogenous reference gene for RT-qPCR normalisation in all samples.

### 2.7. Statistical Analysis

*TSPAN32* relative expression (2^−ΔΔCt, Livak method) was calculated for each patient using the pre-chemoradiation sample as the intra-patient calibrator. Because the pre-treatment value is fixed at ΔΔCt = 0 by design, the treatment effect across the ten paired patients was evaluated using a one-sample *t*-test on the log2-transformed fold-change (Log_2_FC) values, testing the null hypothesis that mean Log_2_FC = 0. Normality was assessed with the Shapiro–Wilk test. A Wilcoxon signed-rank test was performed as a non-parametric confirmatory analysis. Statistical significance was defined as *p* < 0.05. All statistical analyses were performed using IBM SPSS Statistics version 20 and JASP. Kaplan–Meier survival curves were generated using KM-Plotter and log-rank test *p*-values and hazard ratios with 95% CIs are reported.

## 3. Results

### 3.1. Subject Characteristics

The demographic and clinical characteristics of ten nasopharyngeal carcinoma (NPC) patients included in this study are summarized in Table 1. The mean age of the cohort was 52.1 ± 9.6 years, ranging from 36 to 70 years. Five patients (NPC 01, 02, 03, 08, 09) received red blood cell transfusions with volumes between 204 mL and 438 mL, while five patients did not undergo transfusion. Most cases were classified as stage III or IV according to TNM and WHO staging, indicating advanced disease at presentation. Two patients (NPC 04 and NPC 06) exhibited distant metastasis (M1), corresponding to stage IVc. All patients completed the planned chemoradiation course. RNA quality control confirmed acceptable purity (A260/A280: 1.8–2.0) and sufficient concentration (≥10 ng/μL) across all paired samples.

### 3.2. DEG Identification from GSE53819

**Figure 1:**
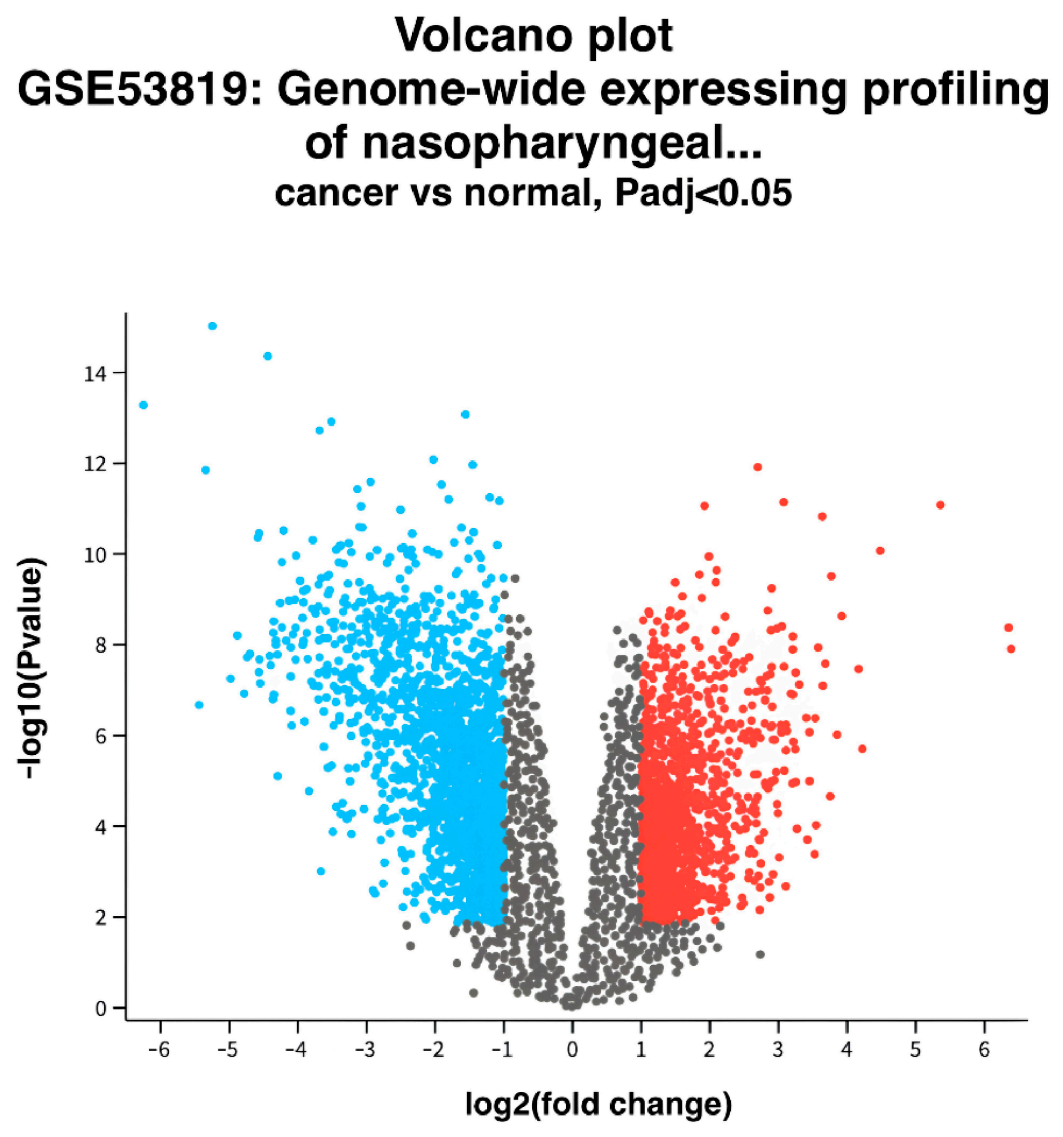
Volcano plot of all DEGs from datasets GSE53819. Red dots visualized upregulated DEGs (log_2_FC > 1) and blue dots visualized downregulated DEGs (log_2_FC < −1). All the colored dots were statically significant (pAdj < 0.05).

Analysis of the GSE53819 microarray dataset (18 advanced-stage NPC tissues vs. 18 non-cancerous nasopharyngeal tissues) identified a total of 2446 differentially expressed genes (DEGs) meeting the predefined thresholds (|log_2_FC| ≥ 1; Padj < 0.05), comprising 943 upregulated and 1503 downregulated genes. The distribution of DEGs is visualized in the volcano plot (Figure 1).

### 3.3. PPI Network and Functional Enrichment Analysis

**Figure 2:**
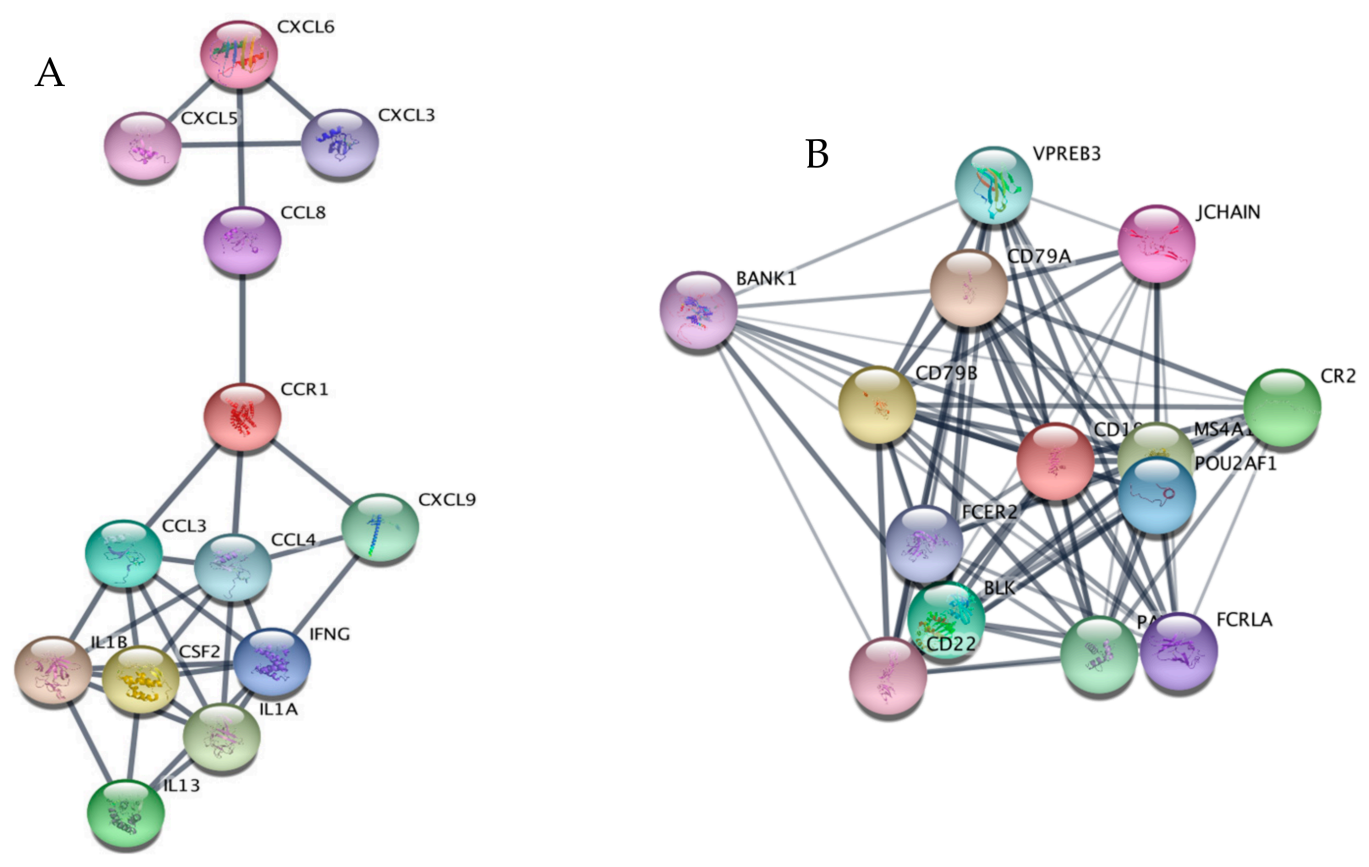
PPI network of advanced-stage nasopharyngeal cancer (visualized in Cytoscape software version 3.8.2): (**A**). Upregulated DEGs and (**B**). Downregulated DEGs.

PPI network construction and hub gene identification using the MCC algorithm revealed two distinct interaction clusters: an upregulated network dominated by chemokine and cytokine-associated proteins (CXCL3, CXCL5, CXCL6, CXCL9, CCL3, CCL4, CCL8, CCR1, CSF2, IFNG, IL1A, IL1B, IL13), and a downregulated network enriched in B-cell signalling components (CD19, CD22, CD79A, CD79B, BLK, BANK1, FCRLA, JCHAIN, CR2, MS4A1, VPREB3, FCER2, POU2AF1) (Figure 2).

**Figure 3:**
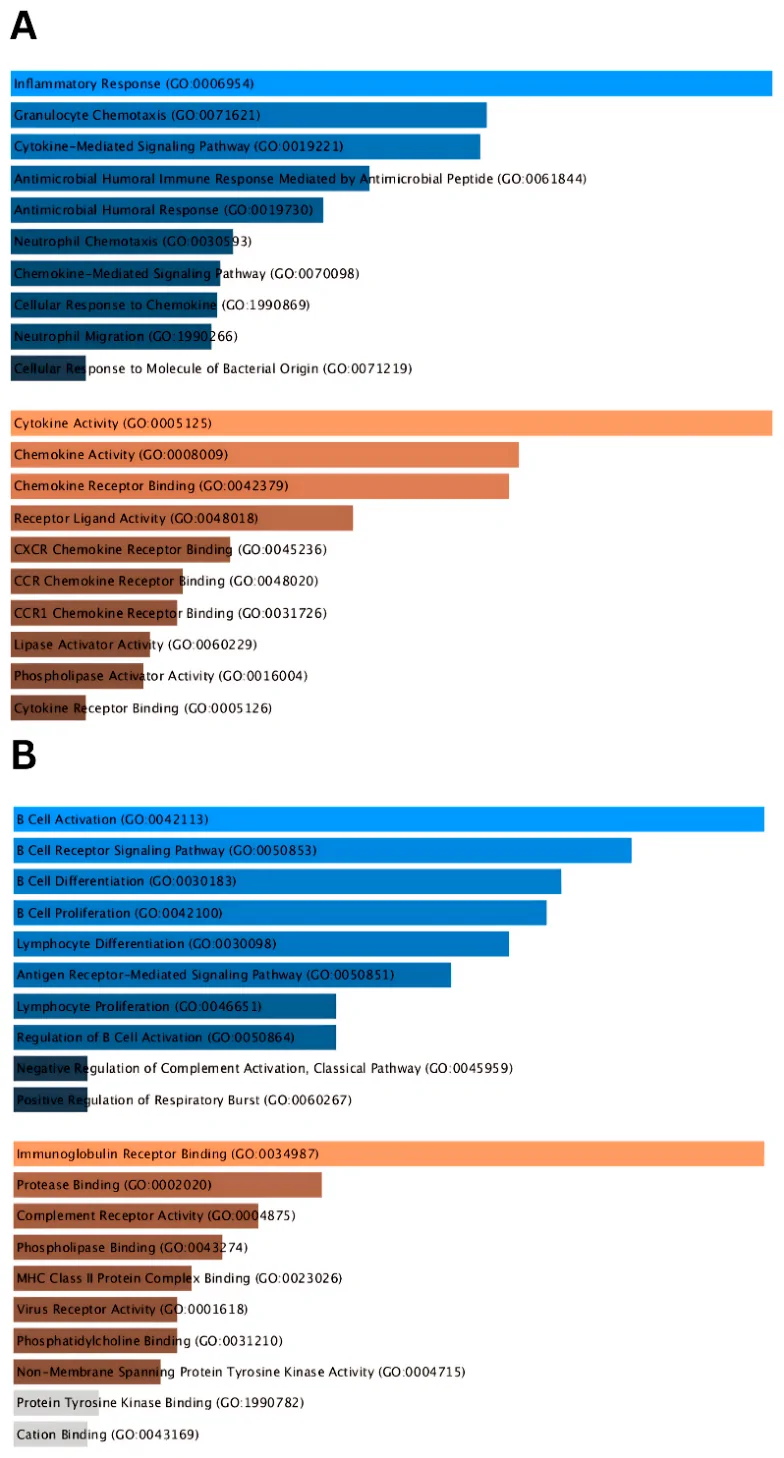
Gene ontology of advanced-stage nasopharyngeal cancer (visualized using Enrichr): (**A**). Upregulated DEGs and (**B**). Downregulated DEGs. Blue bar = Biological Process (BP), Brown bar = Molecular Function (MF).

GO enrichment analysis confirmed that upregulated DEGs were predominantly enriched in inflammatory response (Biological Process, BP) and cytokine activity (Molecular Function, MF), while downregulated DEGs were most significantly enriched in B-cell activation (BP) and immunoglobulin receptor binding (MF) (Figure 3).

**Figure 4:**
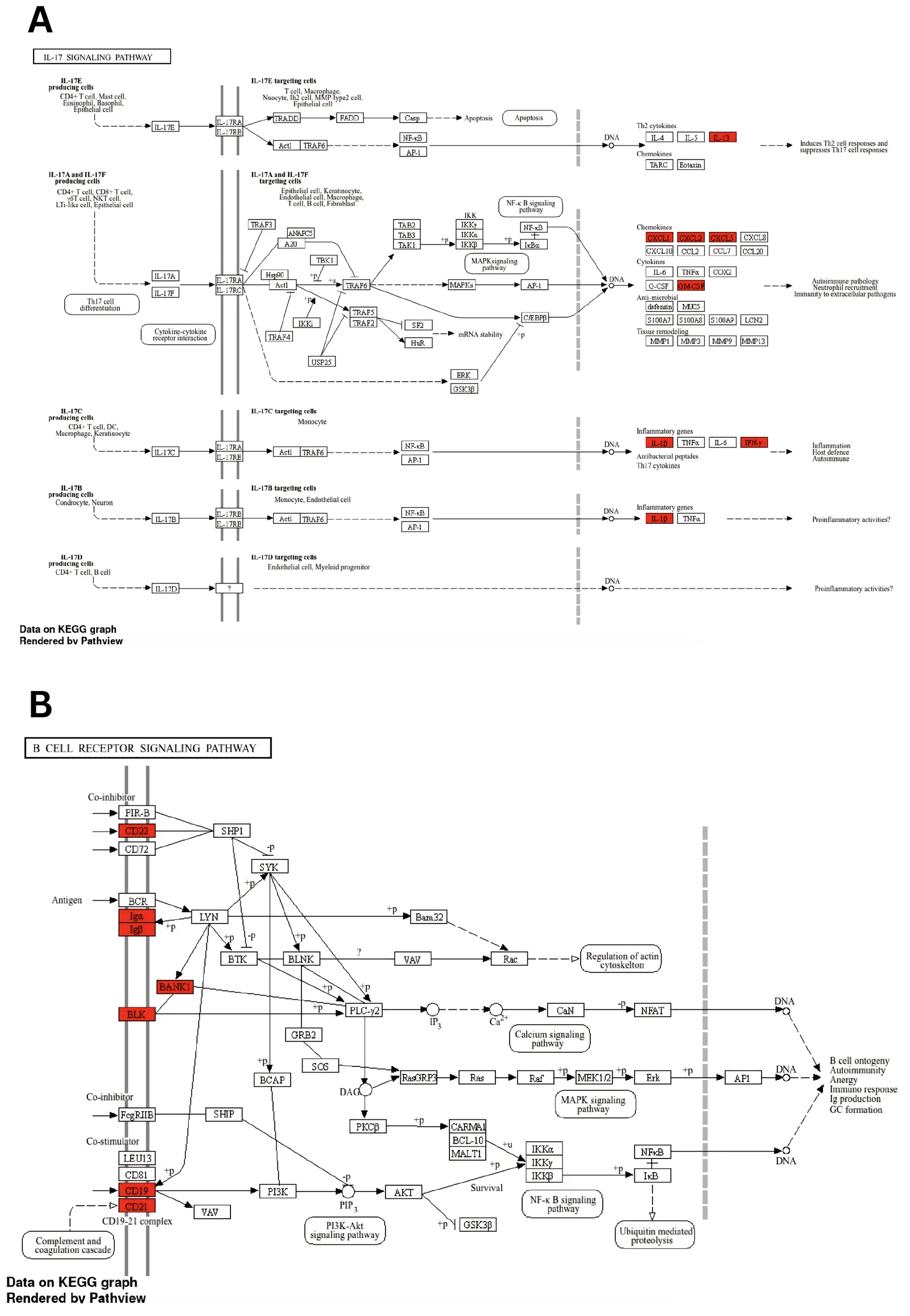
KEGG pathway of advanced-stage nasopharyngeal cancer (visualized using ShinyGO v.0.82). Solid arrows indicate direct molecular interactions (e.g., activation or binding), dashed arrows indicate indirect relations occurring through unlisted intermediate steps. Lines terminating in an open circle denote an expression relation, indicating downstream transcriptional activation of the listed genes. Red-shaded boxes represent genes identified as significantly upregulated/downregulated DEGs mapped onto the reference pathway; unshaded boxes represent canonical pathway components not identified as significantly altered in this dataset. (**A**). Upregulated DEGs and (**B**). Downregulated DEGs.

KEGG pathway analysis demonstrated that the most enriched pathway for upregulated DEGs was IL-17 signalling, while downregulated DEGs were primarily associated with the B-cell receptor (BCR) signalling pathway (Figure 4). These findings indicate that advanced-stage NPC is characterised by a paradoxical immune state which encompasses heightened pro-inflammatory cytokine activity alongside profound suppression of B-cell-mediated adaptive immunity.

### 3.4. Identification of Candidate Blood-Based Biomarkers

**Figure 5:**
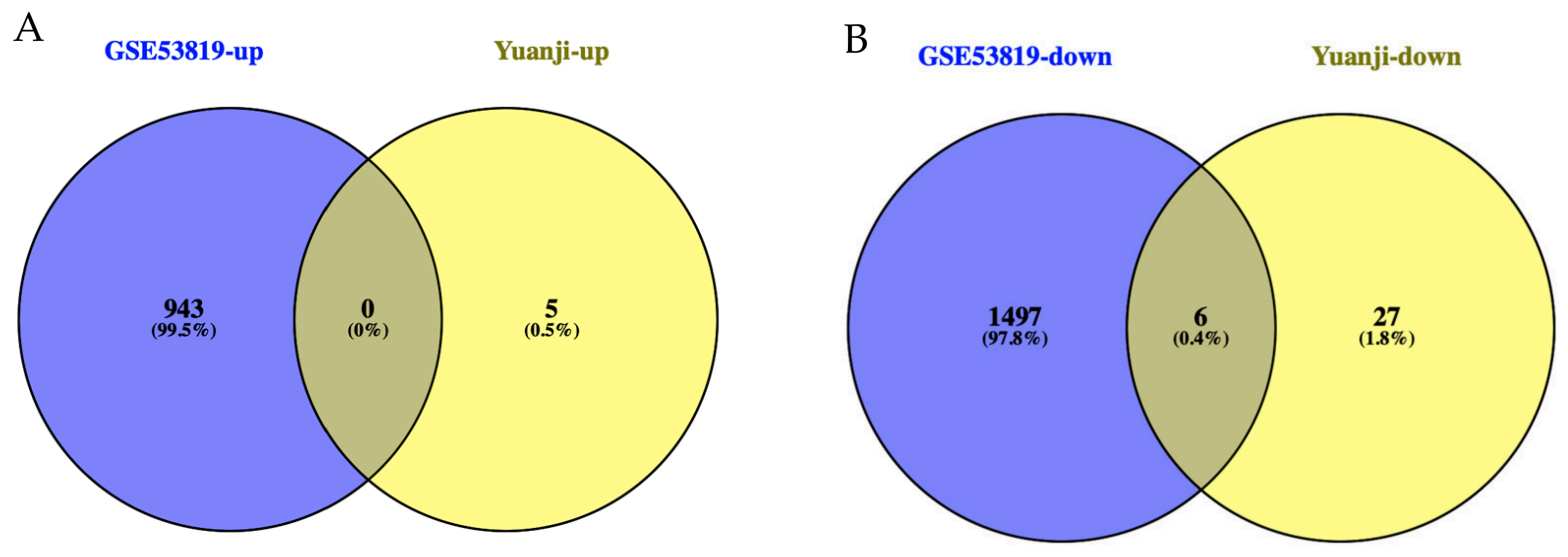
Venn diagram of DEGs found in GSE53819 datasets (tissue samples from NPC biopsy; blue) and Yuanji Xu et al. datasets (blood samples from NPC patients; yellow) [24]: (**A**). Upregulated DEGs and (**B**). Downregulated DEGs.

Cross-referencing of GSE53819 downregulated DEGs with the blood-based NPC transcriptomic dataset of Yuanji Xu et al. [24] identified six concordantly downregulated genes across both the tissue and peripheral blood compartments: *ADRA2A*, *SELP*, *TSPAN32*, *PEAR1*, *P2RX1*, and *DGKG* (Figure 5). No upregulated genes were concordantly identified in both compartments. These six genes represent candidates whose expression in peripheral blood mirrors the tissue-level downregulation observed in advanced-stage NPC, making them conceptually accessible as minimally invasive circulating biomarkers.

### 3.5. Survival Analysis

**Figure 6:**
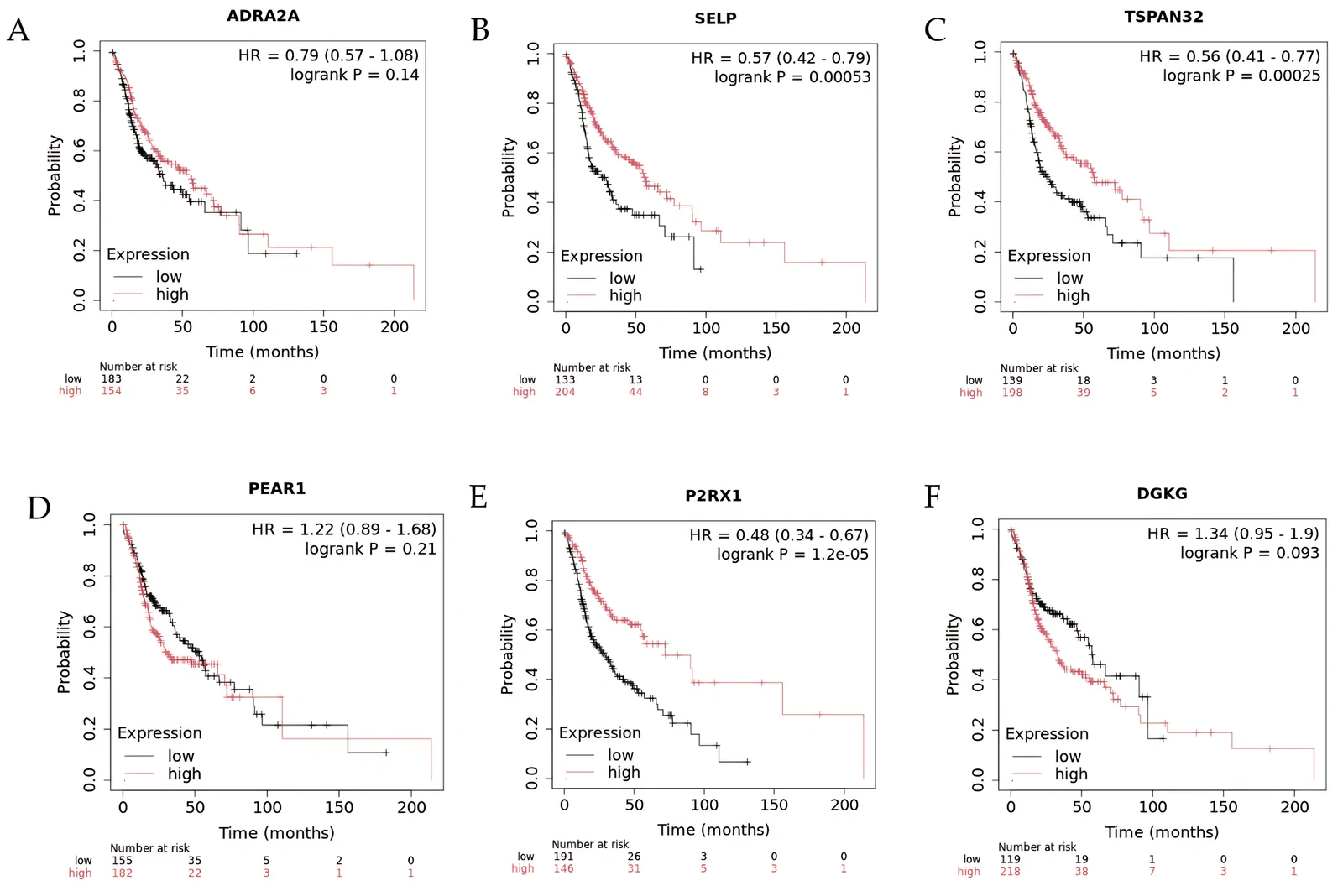
Kaplan–Meier plot of 6 overlapped DEGs of advanced-stage NPC: (**A**). *ADRA2A* gene, (**B**). *SELP* gene, (**C**). *TSPAN32* gene, (**D**). *PEAR1* gene, (**E**). *P2RX1* gene, and (**F**). *DGKG* gene.

Kaplan–Meier survival analysis of the six overlapping candidate DEGs revealed that lower expression of three genes was significantly associated with shorter overall survival in head and neck carcinoma: *SELP* (HR = 0.57, 95% CI 0.42–0.79; log-rank *p* = 0.00053), *TSPAN32* (HR = 0.56, 95% CI 0.41–0.77; log-rank *p* = 0.00025), and *P2RX1* (HR = 0.48, 95% CI 0.34–0.67; log-rank *p* = 1.2 × 10^−5^). *ADRA2A* (HR = 0.79, *p* = 0.14), *PEAR1* (HR = 1.22, *p* = 0.21), and *DGKG* (HR = 1.34, *p* = 0.093) did not reach statistical significance (Figure 6). The hazard ratios below 1.0 for *SELP*, *TSPAN32*, and *P2RX1* indicate that patients with lower expression of these genes had shorter overall survival, affirming the prognostic relevance of their downregulation in advanced-stage disease.

*TSPAN32* was prioritized as the primary candidate for RT-qPCR examination based on: (i) a statistically significant and consistent survival association among the three significant candidates, with an effect size (HR = 0.56) comparable to *SELP* and *P2RX1;* (ii) its established functional role in B-cell activation and immune regulation, mechanistically coherent with the B-cell pathway suppression identified in the enrichment analyses; and (iii) prior published evidence of *TSPAN32* downregulation in peripheral immune cell populations in inflammatory and immune-dysregulation [40].

### 3.6. TSPAN32 Primer Design

Primers for RT-qPCR quantification of *TSPAN32* were designed and quality-validated as described in the Methods. The primer sequences, product size, and thermodynamic parameters are presented in Table 2. No hairpin or primer-dimer formation was predicted, confirming primer suitability for downstream RT-qPCR assays.

### 3.7. TSPAN32 Expression in Peripheral Blood Following Chemoradiation

**Figure 7:**
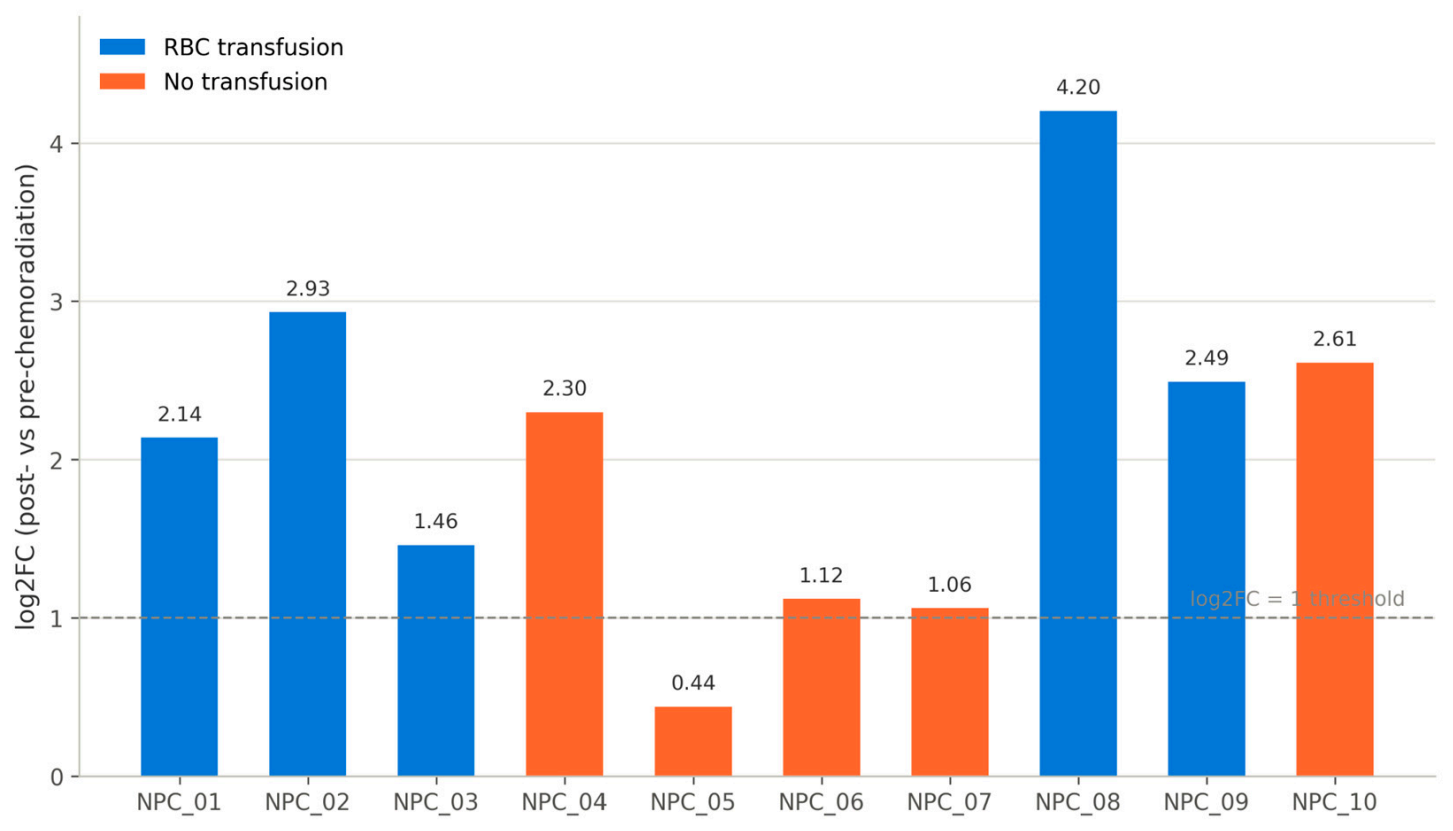
The *TSPAN32* expression changes in peripheral blood following chemoradiation among patients (*n* = 10).

Across the ten paired peripheral blood samples, *TSPAN32* transcript abundance increased following chemoradiation relative to each patient’s own pre-treatment baseline (mean Log_2_FC = 2.08 ± SD 1.09; 95% CI: 1.29 to 2.86) (Figure 7). Nine of ten patients (90%) showed a fold-change consistent with upregulation (Log_2_FC > 1); one patient (Patient 5, Log_2_FC = 0.44) did not meet this threshold (Table 3). This increase was statistically significant by one-sample *t*-test (t(9) = 6.01, *p* = 0.0002) and confirmed by Wilcoxon signed-rank test (*p* = 0.002). The melt-curve analysis for each patient was presented in Figure S1.

### 3.8. Effect of RBC Transfusion on TSPAN32 Expression Change

As a secondary analysis, the effect of RBC transfusion on the magnitude of *TSPAN32* expression change was assessed within the 10 patients clinical cohort (Table 4). The mean post-treatment log_2_FC was numerically higher in the transfusion group (n = 5; mean 2.64 ± 1.02) than in the non-transfusion group (*n* = 5; mean 1.51 ± 0.91), but this difference did not reach statistical significance (independent-samples *t*-test, t = 1.86, *p* = 0.101). Post-treatment relative expression likewise did not differ significantly between groups (Mann–Whitney U test, exact, U = 20.0, *p* = 0.151). These findings suggest that RBC transfusion during chemoradiation did not significantly modulate the magnitude of *TSPAN32* expression change in this cohort, indicating that the observed post-chemoradiation upregulation of *TSPAN32* is attributable primarily to treatment effects rather than transfusion-associated immune modulation.

## 4. Discussion

Effective and suitable biomarkers for the early detection, diagnosis, and prognosis of NPC remain notably few [24,41]. The identification of reliable molecular biomarkers that are detectable in both tissue and peripheral blood remains an important and critical unmet need in NPC clinical management. While the advancement of high-throughput techniques, especially large-scale whole-genome sequencing and RNA sequencing, and the molecular signatures of NPC, including genomic alterations, susceptibility genes, gene expression patterns (both in host and EBV), host–virus interactions, and transcriptional features, have already been unveiled [42], proteins and transcripts that are concordantly dysregulated across both tissue and liquid biopsy compartments offer particular translational value as minimally invasive progression markers. Therefore, this study presents an integrative approach to circulating biomarker discovery in advanced-stage NPC, combining *in silico* transcriptomic analysis of a publicly available tissue dataset with cross-referencing against a blood-based NPC transcriptomic dataset and prospective RT-qPCR examination in peripheral blood.

Of the total patients diagnosed with NPC and undergoing chemoradiation at the hematology oncology outpatient clinic at Cipto Mangunkusumo Hospital, 10 NPC patients, consisting of 6 men (60%) and 4 women (40%), with an average age of 52.1 ± 9.6 years, ranging from 36 to 70 years. The observed male predominance is consistent with the well-established epidemiological pattern of NPC globally and in the Southeast Asian region. Several prior Indonesian studies have reported analogous sex distributions [1,43,44] and a large international surveillance encompassing 20 regions worldwide has documented that men experience approximately a three-fold higher incidence of NPC compared to women (2.2 vs. 0.8 cases per 100,000 person-years) [45]. The biological mechanisms underlying this disparity likely involve differences in EBV seroprevalence, hormonal modulation of immune responses, and occupational or lifestyle exposures that disproportionately affect men [46,47].

Differential gene expression analysis of the tissue-derived DEGs from GSE53819 successfully identified several upregulated as well as downregulated genes. The analysis of the GSE53819 dataset yielded 943 upregulated and 1503 downregulated DEGs under the same threshold criteria. Hierarchical clustering analysis consistently demonstrated that advanced-stage NPC was characterised by substantially greater transcriptomic deviation from the healthy baseline than early-stage disease, indicating that progressive molecular dysregulation accompanies tumour advancement. Functional enrichment via the GO and KEGG databases highlighted that upregulated DEGs were predominantly enriched in inflammatory response pathways (Biological Process, BP) and cytokine activity (Molecular Function, MF), with the interleukin (IL)-17 signalling pathway emerging as the most enriched KEGG pathway. This finding is consistent with other studies showing that bioinformatic studies on NPC utilizing the GEO dataset found IL-17 as one of the signaling pathways enriched in KEGG [48,49,50]. IL-17 is a pro-inflammatory cytokine produced by T helper cells (Th17) and has several subsets, namely IL-17A-F [29,51]. IL-17 has several roles in both acute and chronic host defense responses [31,52].

In a pathological context, IL-17 is a primary cytokine and therapeutic target in a number of infectious, autoimmune, inflammatory diseases, and cancers [51,52,53]. IL-17 signaling can activate the downstream NF-kB p76 pathway, a key mediator of NF-kB signaling, which triggers the production of inflammatory factors such as IL-1β, IL-6, and TNFα, thereby promoting inflammation [53]. In NPC, immune microenvironment studies have focused on T cells, which are known to undergo changes in response to EBV infection. IL-17 has been reported to promote the accumulation of polymorphonuclear myeloid-derived suppressor cells (PMN-MDSCs), while neutrophil-derived IL-17 also suppresses CD8+ T cell responses, both contributing to immunosuppression [54]. In addition, the cytokine IL-17, especially IL-17A, is known to play an important role in promoting NPC cell migration and invasion through the p38 mitogen-activated protein kinase/NF-κB signaling pathway, which subsequently upregulates the expression of metallopeptidase (MMP) 2 and MMP9 and increases epithelial–mesenchymal transition (EMT) [51,55]. Downregulated DEGs were most significantly enriched in B-cell activation (BP) and immunoglobulin receptor binding (MF), with the B-cell receptor signalling pathway as the corresponding KEGG pathway. This aligns with previous study findings demonstrating that B cell depletion is a hallmark feature of microenvironmental conditions exhibited in NPC progression [56]. This is clinically relevant given that high B cell infiltration, particularly naïve B cells, is associated with a better prognosis in NPC [57]. Therefore, the downregulation of B cell-related DEGs we identified in advanced stages may reflect the loss of this protective B cell population, which in turn contributes to immune evasion and tumor progression.

Cross-validation the GSE53819 tissue-based DEGs with the blood-based DEGs identified by Yuanji Xu et al. [24] revealed six concordantly downregulated genes across both compartments: *ADRA2A*, *SELP*, *TSPAN32*, *PEAR1*, *P2RX1*, and *DGKG*. The Kaplan–Meier survival analysis provides important prognostic context for these six candidate DEGs. The significant association between lower *TSPAN32* expression and shorter overall survival (HR = 0.56; *p* = 0.00025) establishes prognostic relevance beyond the transcriptomic signature alone, and supports the hypothesis that *TSPAN32* downregulation is not merely an epiphenomenon of tumour transcriptomics but reflects a biologically meaningful alteration in the immune microenvironment with consequences for disease outcome. The similar associations observed for *SELP* and *P2RX1* suggest that the identified six-DEG cluster may represent a network of functionally related immune regulatory genes that are collectively dysregulated as NPC progresses, and whose combined suppression is associated with adverse clinical outcomes. However, this analysis is based on a general head and neck carcinoma dataset, not an NPC-specific dataset, so interpretation of the prognosis from *TSPAN32* should be done with caution.

Based on the cross-validation results, *TSPAN32* was prioritised as the candidate biomarker of most clinical interest [40,58]. TSPAN32 (also known as TSSC6) is a tetraspanin-family member encoded within the imprinted tumor-suppressor gene domain on chromosome 11p15.5, where it plays a role in the formation of membrane microdomains regulating cell migration, adhesion, signal transduction, and immune responses. It is preferentially expressed in lymphoid-lineage cells, including CD8+ T cells and natural killer cells, and its expression is downregulated upon T-cell activation, consistent with a negative regulatory role in lymphocyte proliferation and immune activation; *TSPAN32*-knockout models show normal hematopoiesis and B-cell responses but hyperproliferative T cells, further supporting an immunoregulatory function [59,60]. Beyond this immunological role, a study evaluating *TSPAN32* as a biomarker associated with radiotherapy and immune microenvironment remodeling in lung adenocarcinoma showed that *TSPAN32* was significantly downregulated, with low expression associated with more advanced stages and poor prognosis, suggesting its tumor-suppressive role. Overexpression of *TSPAN32* inhibited migration, growth, clonogenic survival, and increased radiosensitivity in A549 and H1299 cells. Furthermore, *TSPAN32* expression was also associated with immune/stromal scores and the abundance of T cells, B cells, and immune checkpoints. Notably, *TSPAN32* overexpression promoted infiltration of CD8+ T cells, memory B cells, and dendritic cells [61]. This suggests a possible mechanistic link between *TSPAN32* expression, radiotherapy response, and anti-tumor immune activity that may be relevant to the chemoradiation-associated expression change observed in the present study. However, to our knowledge, no prior study has examined *TSPAN32* expression, prognostic value, or functional role specifically in NPC tissue or peripheral blood. The present findings therefore represent the first report identifying *TSPAN32* as a candidate marker in this malignancy, and its mechanistic role in NPC pathogenesis remains to be functionally established.

RT-qPCR examination using primers designed for the *TSPAN32* gene demonstrated a predominant trend of increased expression of *TSPAN32* after treatment, with 9 out of 10 patients (90%) showing a log_2_ fold change (log_2_FC) greater than 1, indicating clearly elevated expression levels following therapy. One patient (10%) showed a mild positive change (log_2_FC between 0 and 1), and no patient exhibited a negative log_2_FC. This finding shows that *TSPAN32* upregulation following treatment was consistent across nearly the entire cohort, with only minor variation in magnitude. This likely elucidates the function of *TSPAN32*, an integral part of a tumor-suppressing gene, wherein enhanced expression of this gene implies improved immune system activity following treatment [59,61]. Additionally, the RBC transfusion history did not significantly affect the magnitude of *TSPAN32* expression change, with a numerically higher mean post-treatment log_2_FC in the transfusion group (2.64 ± 1.02) compared to the non-transfusion group (1.51 ± 0.91), but this difference did not reach statistical significance (independent-samples *t*-test, t = 1.86, *p* = 0.101). This suggests that the observed post-treatment upregulation pattern is attributable primarily to treatment effects rather than transfusion-associated immune modulation or iron loading from RBC transfusion. This finding has practical implications for future biomarker studies which explains if *TSPAN32* expression monitoring is to be incorporated into clinical evaluation of NPC patients, the concurrent administration of RBC transfusion does not appear to substantially confound the interpretation of *TSPAN32* expression dynamics, at least at the transfusion volumes and time observed in this study.

The integrative design of this study which combines tissue-based with blood-based validation through *in silico* and clinical approaches represents a methodological template that addresses a recognized gap in NPC biomarker research. However, several important limitations of this study must be acknowledged. First, the transcriptomic discovery phase relied on a single, relatively small, publicly available microarray dataset (GSE53819; n = 18 per group), chosen specifically for its advanced-stage-matched case composition; other available NPC datasets (e.g., GSE12452, GSE118613) include different stage distributions. Although downstream PPI and survival analyzes confirmed the DEGs, it was not tested against an independent as well as stage-specific NPC transcriptomic cohort. The potential genes identified here, including *TSPAN32*, should be considered proposed rather than confirmed, and future research combining additional stage-specific datasets, as they become available, are required to establish the robustness of this pattern. Second, the tissue-blood overlap examination compared two datasets characterized by different platforms and tissue compartments (tumor tissue versus peripheral blood), thereby diminishing the sensitivity of cross-identification; the blood-based reference dataset was exclusively derived from platelet RNA sequencing, while the RT-qPCR analysis in this study utilized RNA extracted from the buffy coat (leukocyte-enriched) fraction, representing biologically distinct circulating compartments. Third, the RT-qPCR analysis showed a significant treatment-associated increase in *TSPAN32* expression. However, this design cannot exclude the possibility that this increase partly reflects non-specific elevation of circulating transcripts due to chemoradiation-induced tissue necrosis and cell debris release, rather than a *TSPAN32*-specific biological response, since no unrelated control transcript panel or non-destructive comparative condition were included. Fourth, matched tumor tissue was not sampled following chemoradiation in this cohort, as post-treatment biopsy is not part of routine clinical management for advanced-stage NPC treated with definitive chemoradiation; consequently, this study cannot determine whether the observed peripheral blood expression change corresponds to a change at the tumor level, nor establish the tissue of origin of the circulating signal. Fifth, this study did not include a healthy control comparator group in the RT-qPCR examination phase, so the data demonstrate a within-patient, treatment-associated change but do not establish how *TSPAN32* expression in NPC patients compared to a healthy reference range. Future studies should validate candidate gene expression, including *TSPAN32*, through prospective peripheral blood sampling in matched pre- and post-treatment samples, ideally alongside matched tumor tissue, healthy control comparators, and a panel of unrelated control transcripts, to establish whether these candidates reflect a specific, clinically meaningful signal rather than generalised cell turnover.

## 5. Conclusions

This present bioinformatic discovery approach identified six genes that were downregulated in both NPC tissue and peripheral blood, with *TSPAN32* having the strongest predictive correlation with overall survival. These findings provide preliminary evidence that *TSPAN32* is a potential circulating biomarker in advanced-stage NPC, necessitating additional research in larger, longitudinal cohorts to demonstrate its therapeutic utility for treatment response monitoring and prognostic evaluation.

## Figures and Tables

**Table 1:** Characteristics of nasopharyngeal cancer patients * mean (SD) = 52.1 ± 9.6 years.

Sample ID	Sex	Age (Year) *	Stadium	TNM Staging	Transfusion Volume
NPC 01	Male	48	IVb	T1N3M0	438 mL
NPC 02	Male	54	III	T3N2M0	386 mL
NPC 03	Female	70	III	T2N2M0	222 mL
NPC 04	Male	36	IVc	T4N3M1	No
NPC 05	Male	60	III	T3N1M0	No
NPC 06	Female	56	IVc	T4N3M1	No
NPC 07	Female	50	III	T2N2M0	No
NPC 08	Male	44	IVa	T4N1M0	210 mL
NPC 09	Female	58	IVb	T4N3M0	204 mL
NPC 10	Male	45	III	T2N2M0	No

**Table 2:** Designed primers of *TSPAN32* gene.

Primer Name	Primer Sequences	Product Size (bp)	Tm (°C)	Hairpin	Dimer
TSPAN32F	GCTGTGGGAAGAAGTCTCCT	199	59.02	0	0
TSPAN32R	AACCACAGGAAGGAGCTGAA		58.86	0	0

**Table 3:** *TSPAN32* relative expression for per-patient Log_2_FC (post- vs. pre-chemoradiation).

Sample	Mean Relative Expression	log_2_FC
NPC 01	4.42 (SD 3.48)	2.14
NPC 02	7.63 (SD 1.39)	2.93
NPC 03	2.75 (SD 0.94)	1.46
NPC 04	4.93 (SD 3.34)	2.30
NPC 05	1.36 (SD 0.36)	0.44
NPC 06	2.17 (SD 0.35)	1.12
NPC 07	2.08 (SD 1.60)	1.06
NPC 08	18.36 (11.07)	4.20
NPC 09	5.63 (SD 2.89)	2.49
NPC 10	6.09 (SD 2.74)	2.61

**Table 4:** Comparison of Pre-Treatment, Post-Treatment, and Delta Values among groups.

Parameter (Mean ± SD)	Group Category		Test Statistic	*p*
	No Transfusion (*n* = 5)	RBC Transfusion (*n* = 5)		
*TSPAN32* Log_2_FC	1.51 ± 0.91	2.64 ± 1.02	t = 1.86 ^a^	0.101
Post-treatment Mean Relative Expression	3.33 ± 2.06	7.76 ± 6.19	U = 20.0 ^b^	0.151

^a^: Independent sample *t*-test abnormal. ^b^: Mann–Whitney Y test.

## Data Availability

The original contributions presented in this study are included in the article/Supplementary Material. Further inquiries can be directed to the corresponding author.

## Supplementary Materials

The following supporting information can be downloaded at: https://www.mdpi.com/article/10.3390/cimb48101003/s1.

## Abbreviations

The following abbreviations are used in this manuscript:

ASIR	Age-Standardized Incidence Rate
BCR	B-cell Receptor
BP	B-cell Activation Pathway
cDNA	Complementary DNA
DEG	Differentially Expressed Gene
DEM	Differentially Expressed miRNA
EBV	Epstein–Barr Virus
ENT	Ear, Nose, and Throat
EMT	Epithelial–Mesenchymal Transition
GEO	Gene Expression Omnibus
GLOBACAN	Global Cancer Observatory
IMRT	Intensity-Modulated Radiotherapy
log_2_FC	log_2_ fold change
MF	Molecular Function
MMP	Metallopeptidase
NCBI	National Center for Biotechnology Information
NPC	Nasopharyngeal Cancer
OS	Overall Survival
PMN-MDSCs	Polymorphonuclear Myeloid-Derived Suppressor Cells
PPI	Protein–Protein Interaction
RBC	Red Blood Cell
Th17	T Helper Cell
TSPAN32	Tetraspanin 32
