# Supplementary material for: Preliminary Pilot Assessment of TSPAN32 Detectability in Peripheral Blood as a Potential Circulating Biomarker in Advanced-Stage Nasopharyngeal Carcinoma Patients Undergoing Chemoradiation
Source: Curr Issues Mol Biol. 2026 Sep 29;48(10):1003. doi: 10.3390/cimb48101003 (PMC13703889; doi:10.3390/cimb48101003)
Supplement: Supplementary file 1 [file cimb-48-01003-s001.zip › cimb-4537130-supplementary Figure S1.docx.pdf]

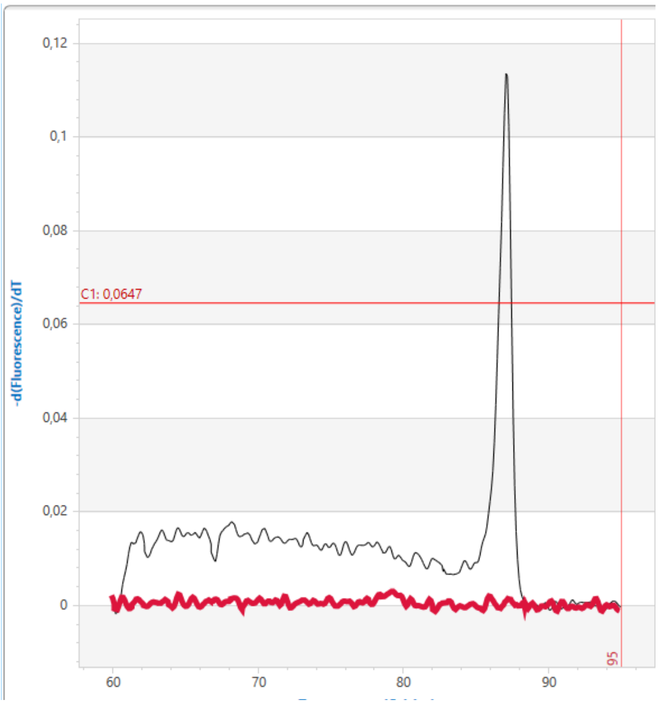

-d(Fluorescence)/dT
0,12
0,1
0,08
0,06
0,04
0,02
0
C1: 0,0647
95
60
70
80
90

1A

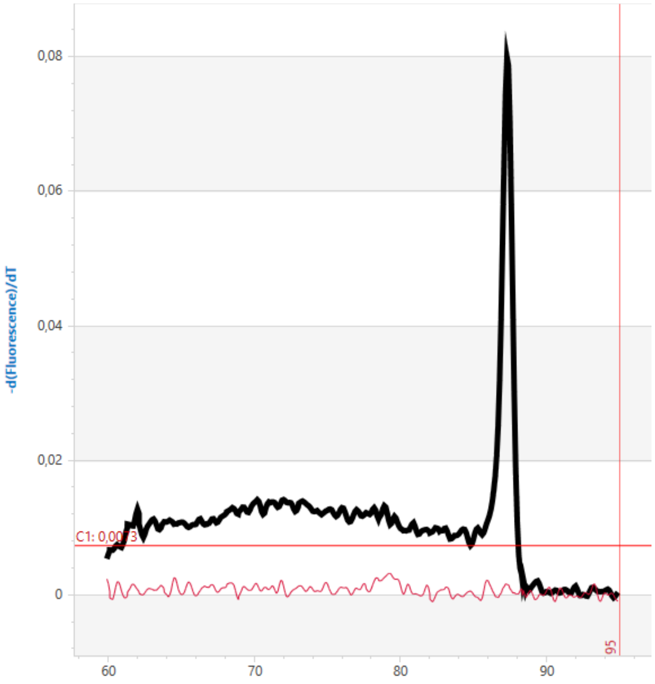

-d(Fluorescence)/dT
0,08
0,06
0,04
0,02
0
95
60
70
80
90

1B

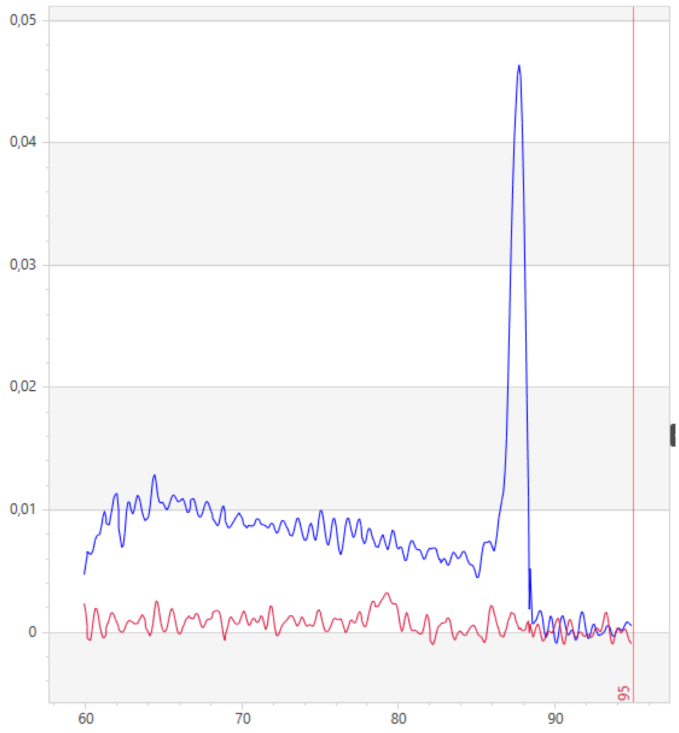
0,05
0,04
0,03
0,02
0,01
0
95
60
70
80
90
2A

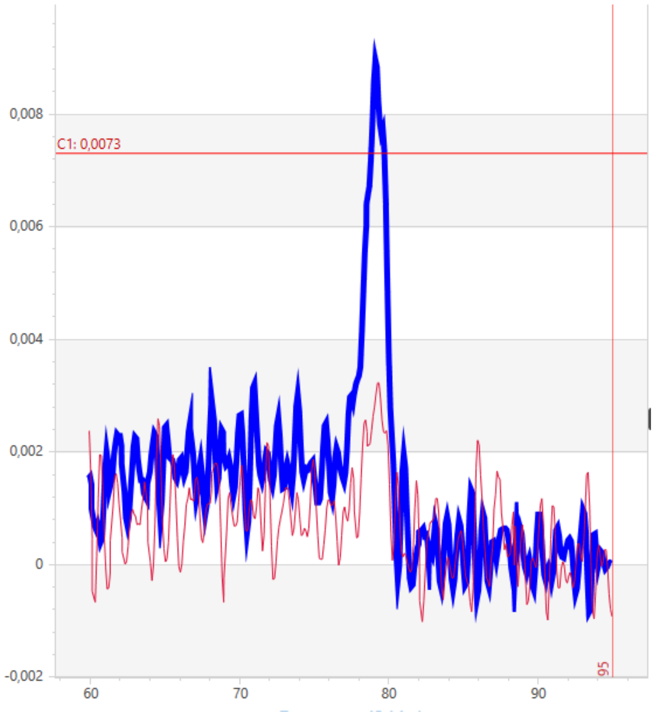
0,008
C1: 0,0073
0,006
0,004
0,002
0
-0,002
95
60
70
80
90
2B

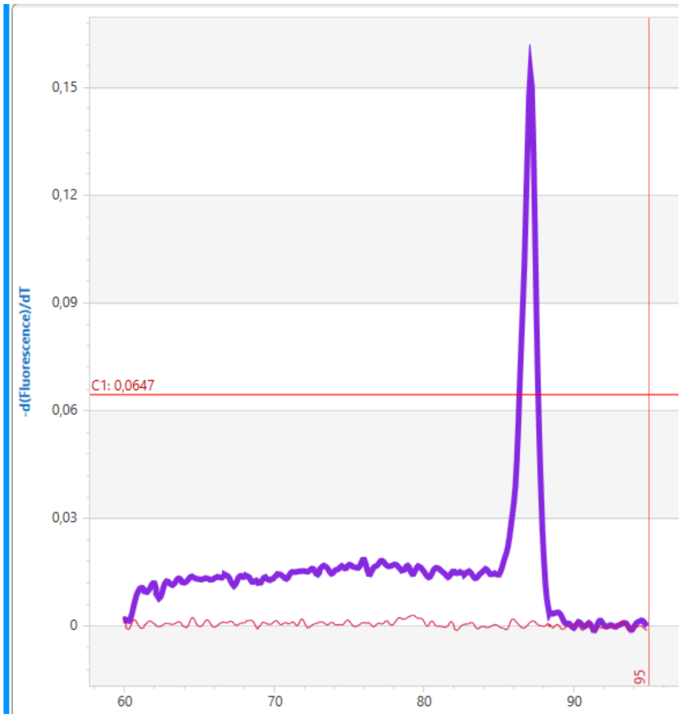
-d(Fluorescence)/dT
0,15
0,12
0,09
C1: 0,0647
0,06
0,03
0
95
60
70
80
90

3A

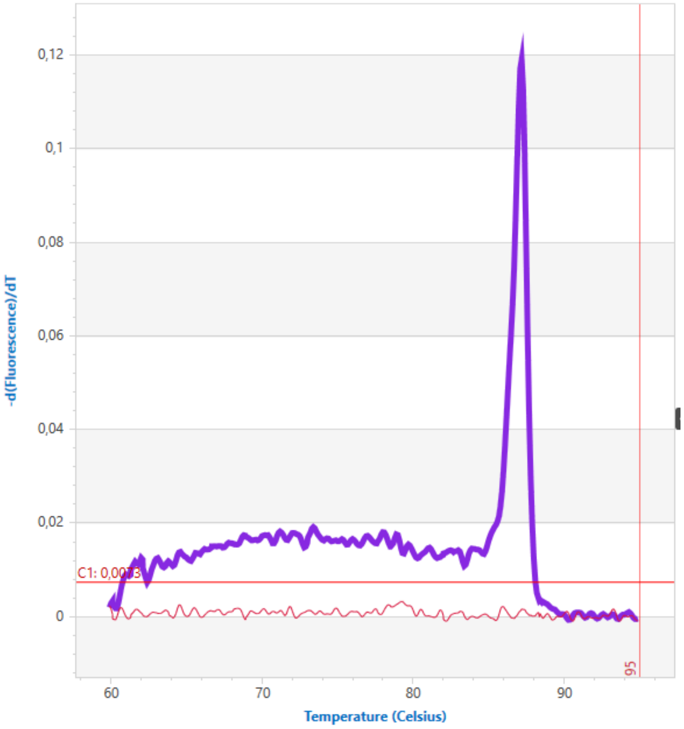
-d(Fluorescence)/dT
0,12
0,1
0,08
0,06
0,04
0,02
C1:
0
95
60
70
80
90
Temperature (Celsius)

3B

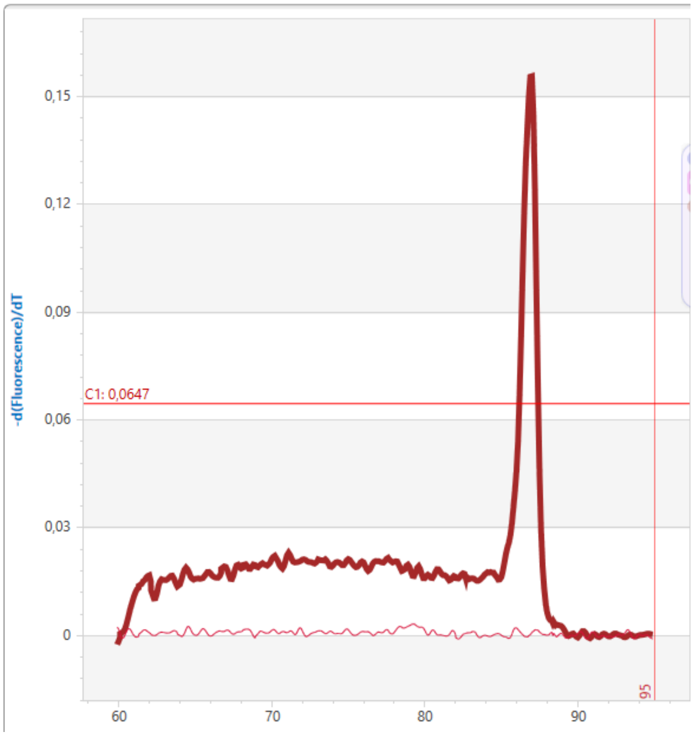
0,15
0,12
0,09
0,06
0,03
0
-d(Fluorescence)/dT
C1: 0,0647
95
60
70
80
90
4A

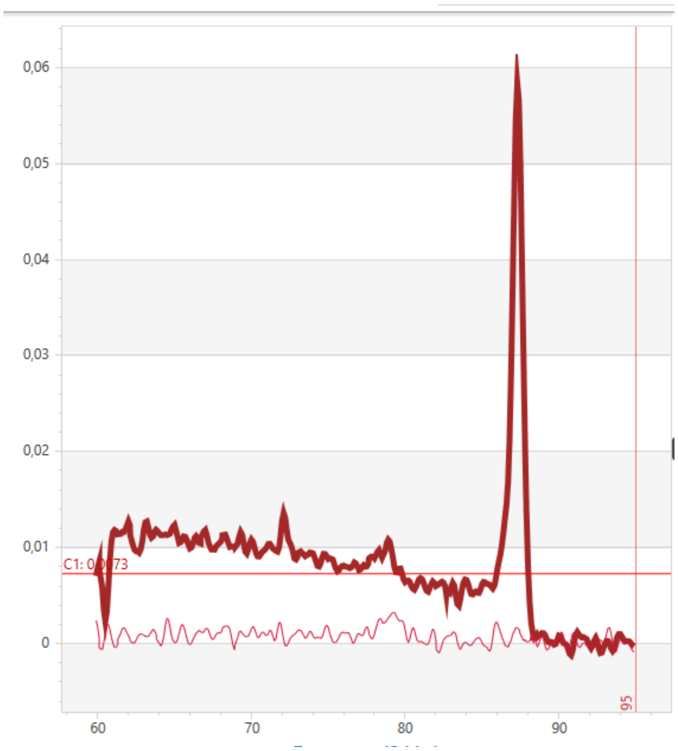
0,06
0,05
0,04
0,03
0,02
0,01
0
95
60
70
80
90
4B

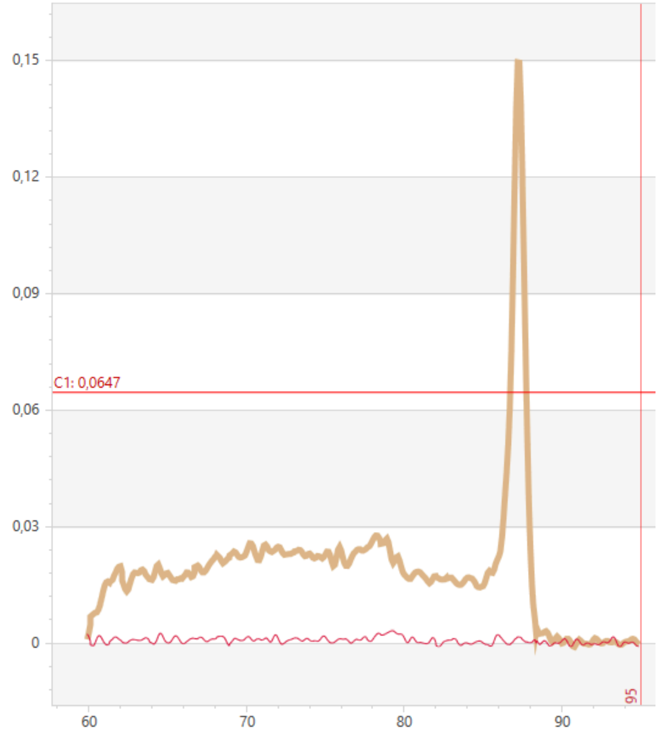
0,15
0,12
0,09
C1: 0,0647
0,06
0,03
0
95
60
70
80
90

5A

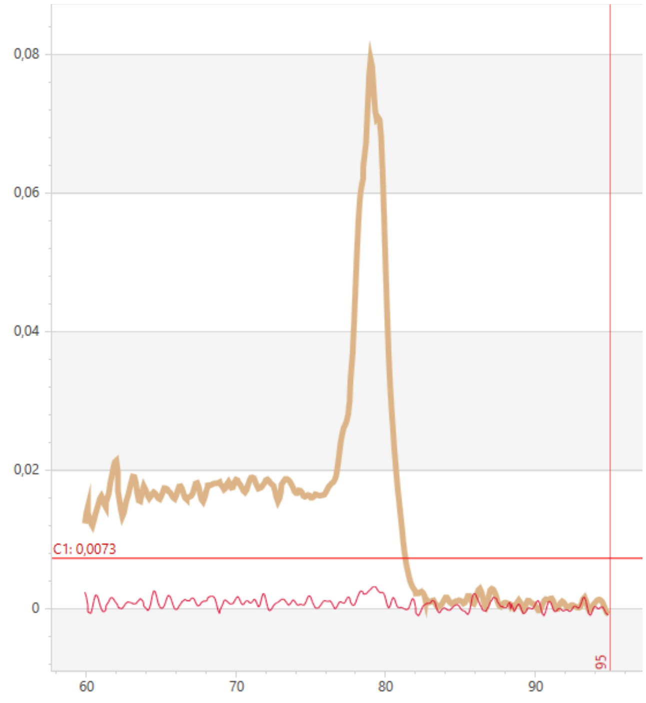
0,08
0,06
0,04
0,02
C1: 0,0073
0
95
60
70
80
90

5B

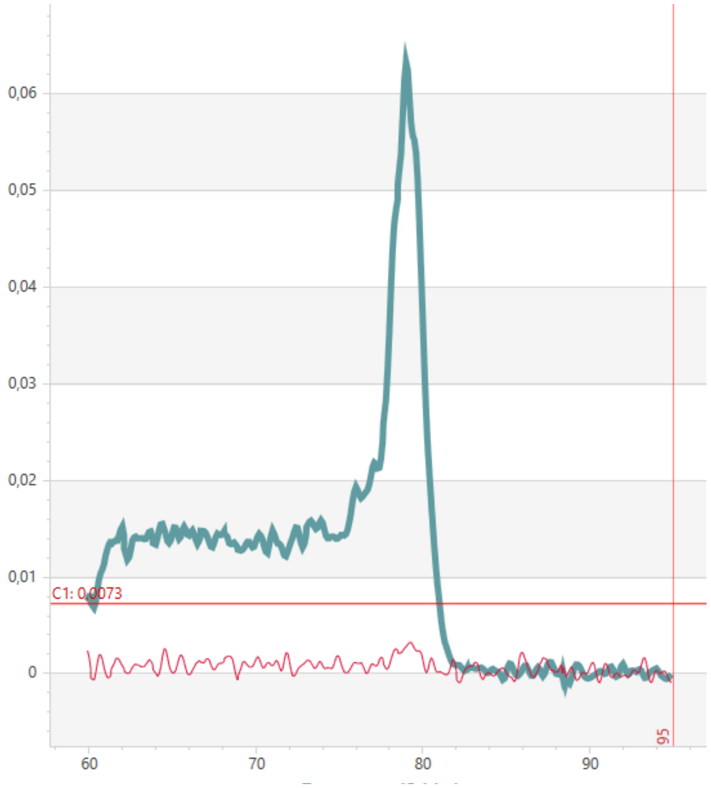
0,06
0,05
0,04
0,03
0,02
0,01
C1: 0,0073
0
95
60
70
80
90

6A

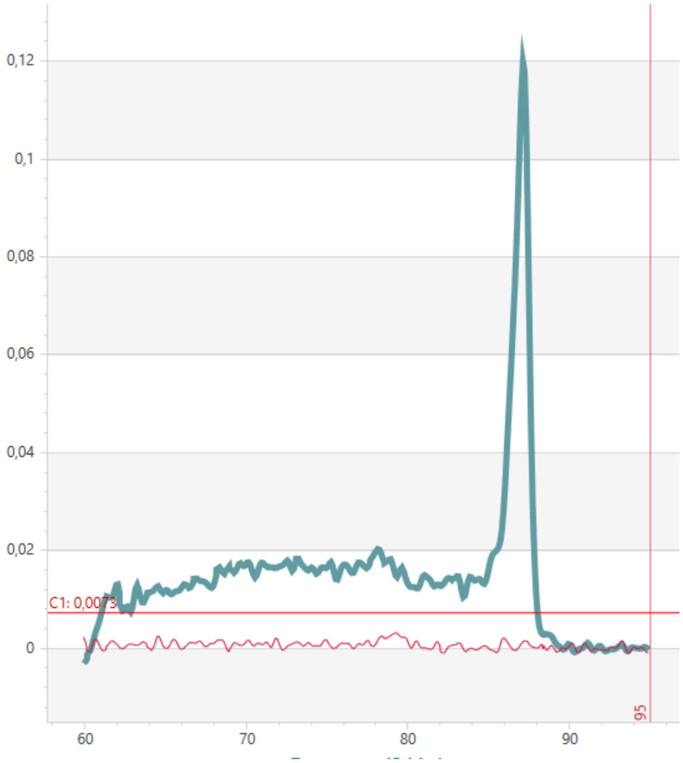
0,12
0,1
0,08
0,06
0,04
0,02
C1: 0,0073
0
95
60
70
80
90

6B

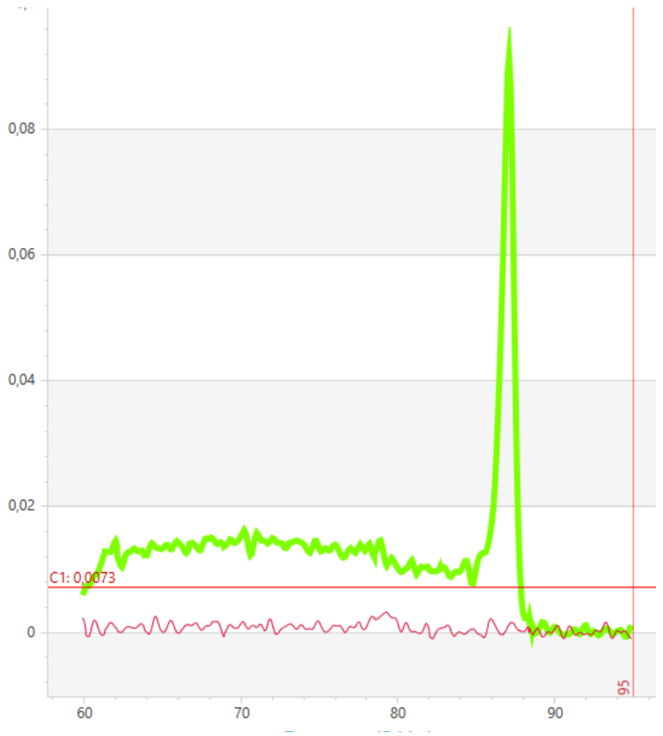
0,08
0,06
0,04
0,02
C1: 0,0073
0
95
60
70
80
90

7A

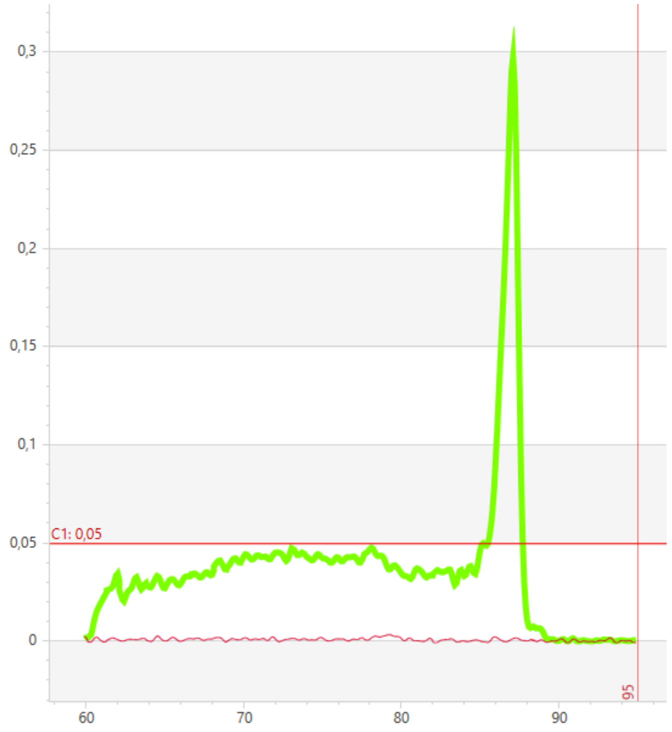
0,3
0,25
0,2
0,15
0,1
C1: 0,05
0,05
0
95
60
70
80
90

7B

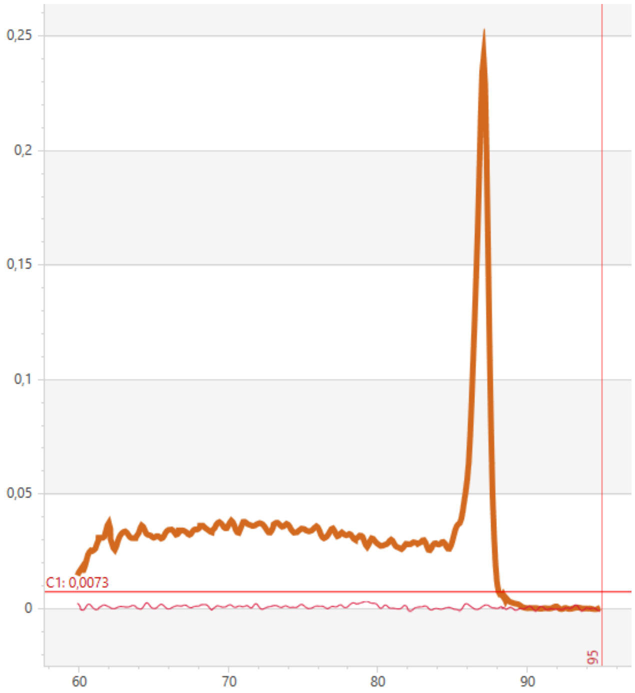
0,25
0,2
0,15
0,1
0,05
0
C1: 0,0073
95
60
70
80
90
8A

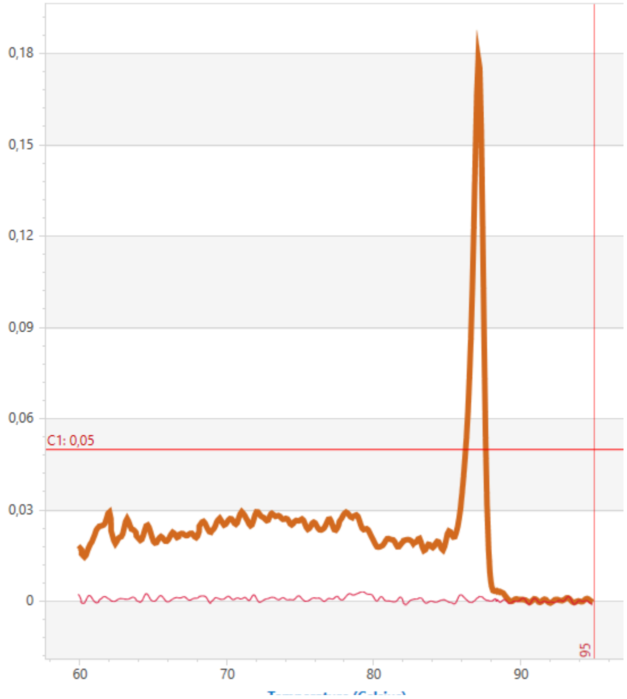
0,18
0,15
0,12
0,09
0,06
C1: 0,05
0,03
0
95
60
70
80
90
8B

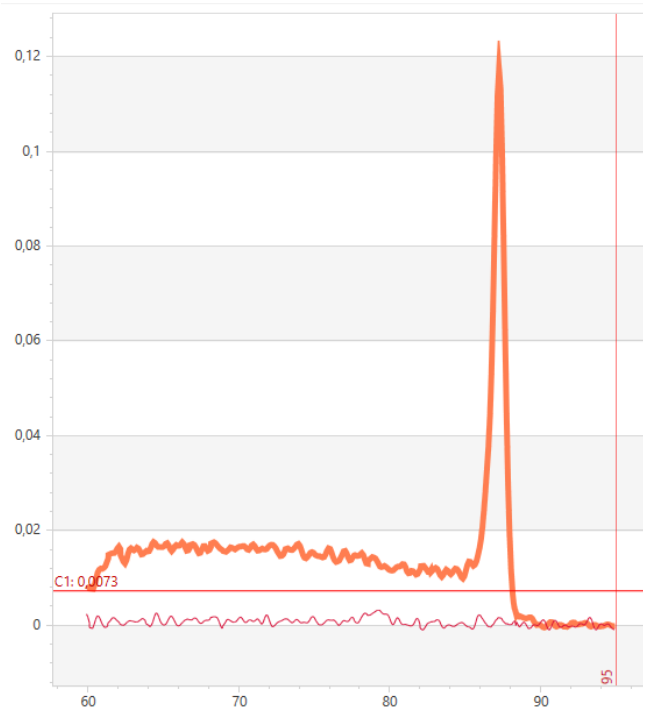
0,12
0,1
0,08
0,06
0,04
0,02
0
C1: 0,0073
95
60
70
80
90

9A

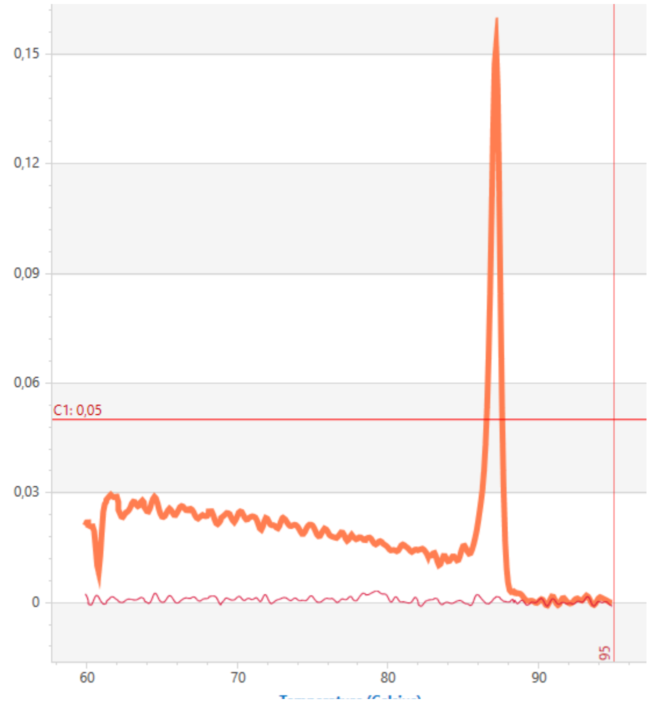
0,15
0,12
0,09
0,06
0,03
0
C1: 0,05
95
60
70
80
90

9B

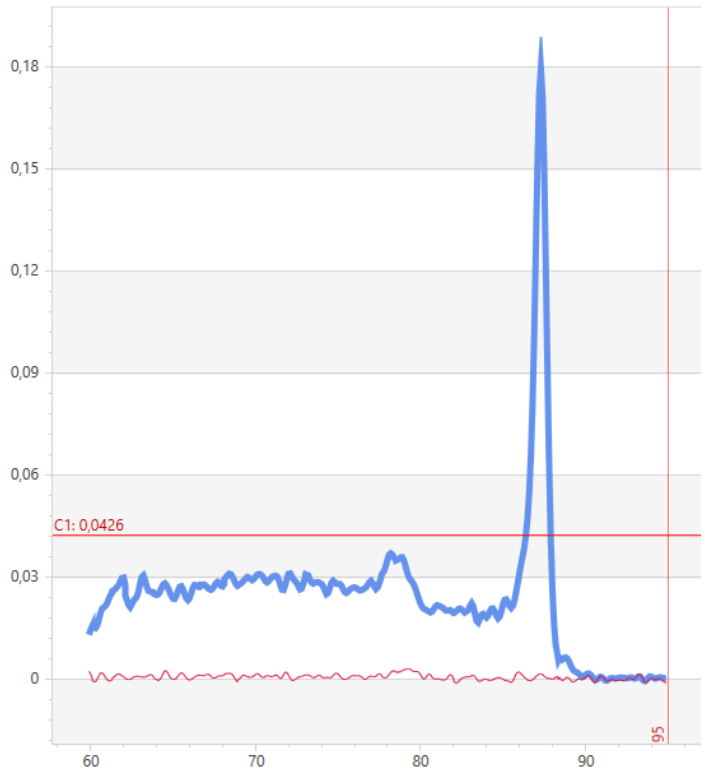

0,18
0,15
0,12
0,09
0,06
C1: 0,0426
0,03
0
95
60
70
80
90

10A

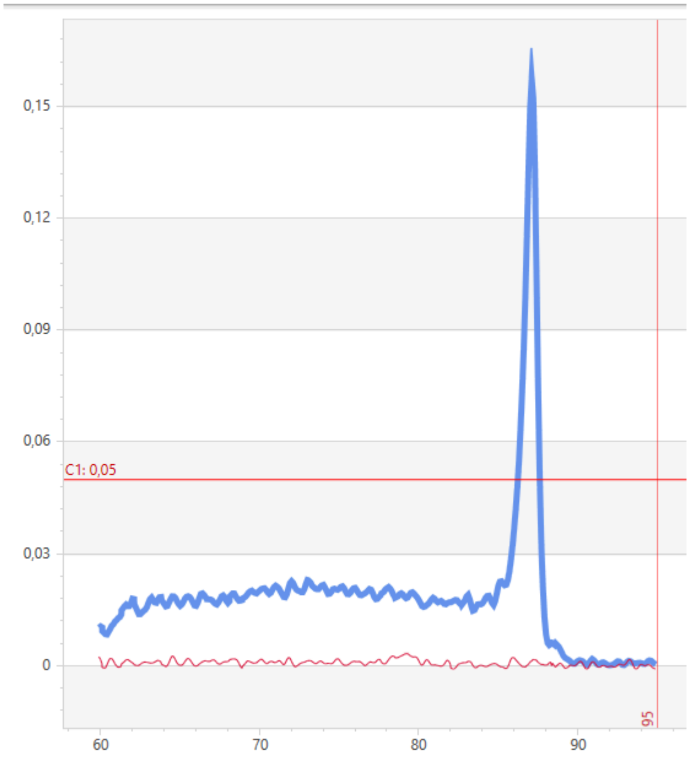

0,15
0,12
0,09
0,06
C1: 0,05
0,03
0
95
60
70
80
90

10B
